# Clinical characteristics and genetic analysis of a Chinese pedigree of type 2 diabetes complicated with interstitial lung disease

**DOI:** 10.3389/fendo.2022.1050200

**Published:** 2023-01-17

**Authors:** Qinghua Zhang, Yan Wang, Chang Tian, Jinyan Yu, Yanlei Li, Junling Yang

**Affiliations:** ^1^ Department of Respiratory and Critical Care Medicine, The Second Hospital of Jilin University, Changchun, China; ^2^ Department of Laboratory Medicine, The Second Hospital of Jilin University, Changchun, China

**Keywords:** type 2 diabetes mellitus, interstitial lung disease, MUC5B, pedigree, whole genome re-sequencing

## Abstract

**Purpose:**

Diabetes mellitus is a systemic metabolic disorder which may target the lungs and lead to interstitial lung disease. The clinical characteristics and mechanisms of type 2 diabetes mellitus (T2DM) complicated with interstitial lung disease (ILD) have been studied. However, little work has been done to assess genetic contributions to the development of T2DM complicated with ILD.

**Method:**

A pedigree of T2DM complicated with ILD was investigated, and the whole genome re-sequencing was performed to identify the genetic variations in the pedigree. According to the literature, the most valuable genetic contributors to the pathogenesis of T2DM complicated with ILD were screened out, and the related cellular functional experiments were also performed.

**Results:**

A large number of SNPs, InDels, SVs and CNVs were identified in eight subjects including two diabetic patients with ILD, two diabetic patients without ILD, and four healthy subjects from the pedigree. After data analysis according to the literature, *MUC5B* SNP rs2943512 (A > C) was considered to be an important potentially pathogenic gene mutation associated with the pathogenesis of ILD in T2DM patients. *In vitro* experiments showed that the expression of MUC5B in BEAS-2B cells was significantly up-regulated by high glucose stimulation, accompanied by the activation of ERK1/2 and the increase of IL-1β and IL-6. When silencing *MUC5B* by RNA interference, the levels of p-ERK1/2 as well as IL-1β and IL-6 in BEAS-2B cells were all significantly decreased.

**Conclusion:**

The identification of these genetic variants in the pedigree enriches our understanding of the potential genetic contributions to T2DM complicated with ILD. *MUC5B* SNP rs2943512 (A > C) or the up-regulated MUC5B in bronchial epithelial cells may be an important factor in promoting ILD inT2DM patients, laying a foundation for future exploration about the pathogenesis of T2DM complicated with ILD.

## 1 Introduction

Diabetes mellitus (DM) is a systemic metabolic disorder characterized by chronic hyperglycemia due to insulin deficiency or resistance (1). The long-term effects of DM include neurological, micro-vascular and macro-vascular complications. The lungs are particularly susceptible targets of diabetic micro-vascular damage and non-enzymatic glycation as a result of their large alveolar-capillary network and abundant connective tissue. Diabetic patients frequently report respiratory symptoms, and diabetes related lung injuries have been observed in several studies (2, 3). Recently, epidemiological studies have suggested that type 2 diabetes (T2DM) is an independent risk factor for interstitial lung disease (ILD) (4–6).

Pulmonary function tests of patients with T2DM show restrictive ventilatory dysfunction and decreased diffusion capacity, including a reduction in forced expiratory volume in one second. High-resolution computed tomography (HRCT) images of the lungs from patients with T2DM are prone to show fibrotic pathological changes, such as a usual interstitial pneumonia pattern (7). However, there is still not enough HRCT data in patients with T2DM to generalize these results. Pathologically, nodular deposition of collagen in the middle of the alveolar walls, and increased thickness of the alveolar epithelial and endothelial capillary basal lamina have been reported to be the features of lung tissue in diabetic patients and animal diabetic models (8–10). A previous study reported significant increases in the cytokines in the bronchoalveolar lavage fluid of patients with T2DM compared to the controls (11), indicating that T2DM could induce inflammation which might promote pulmonary interstitial changes in the lungs. Altogether, clinical and experimental data from numerous studies have revealed that hyperglycemia or T2DM could promote the development of ILD. However, the pathogenic mechanisms involved in the association between T2DM and ILD haven’t been well understood. Previous studies considered unbalanced oxidative stress (12–14), overproduction of advanced glycation end-products (AGEs) and their receptors (15–18), epithelial to mesenchymal transition (EMT) (7, 19, 20), endoplasmic reticulum (ER) stress (21–24), and defects in bronchiolar surfactant layer (25–27) as mechanisms underlying T2DM complicated with ILD. In genetics, abundant variants associated with DM have been identified by genome-wide association studies (GWAS) (28, 29). Additionally, the genetic contributors to the development of ILD have also been identified, especially in cases of familial interstitial pneumonia (FIP) (30). However, it has not yet been determined about the genetic variations accounting for the development of ILD in T2DM.

In this study, a pedigree with T2DM complicated with ILD was investigated, and the whole genome re-sequencing of eight subjects from the pedigree was performed respectively to identify the genetic variations. After the analysis of genetic data based on literature, we proposed a non-synonymous single nucleotide variant (A>C; rs2943512) in *MUC5B* gene or the over-expressed MUC5B in bronchial epithelial cells might be an important factor in promoting ILD in T2DM patients. To our knowledge, this study is the first one presenting potential pathogenic genetic variants in a pedigree of T2DM complicated with ILD.

## 2 Materials and methods

### 2.1 Subject recruitment and information

**Figure 1:**
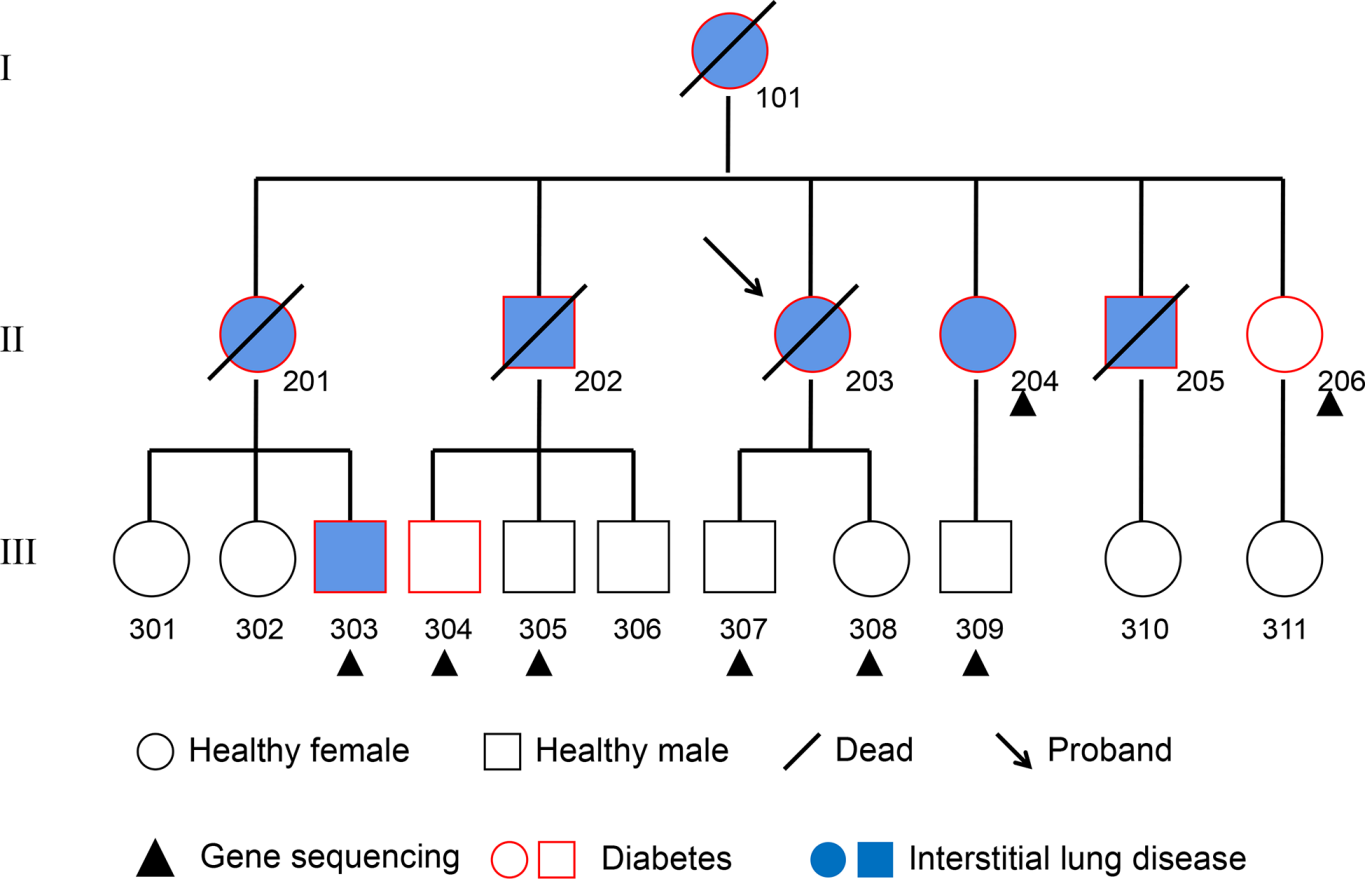
Pedigree Chart. The 8 subjects from the second and third generation who received whole-genome re-sequencing are marked with a black triangle. Squares represent males, circles represent females. The subjects affected by pulmonary fibrosis are painted blue, and the subjects affected by T2DM are indicated with a red border. Deceased individuals are labeled with a black slash.

The subjects in this study were recruited from a pedigree with T2DM and interstitial lung disease in the Jilin Province of China (**Figure 1**). T2DM was diagnosed with the following criteria established by the American Diabetes Association: fasting plasma glucose concentration ≥ 126 mg/dl (7.0 mM), 2-hour post-load plasma glucose ≥200mg/dl (11.1 mM) after the oral glucose tolerance test, history of T2DM and/or on prescribed medication for diabetes. In addition, the tests of antibodies for T1DM were negative in each subject. Interstitial lung disease was collectively diagnosed by two radiologists and three respiratory physicians. Inclusion criteria for the subjects were as follows: 1) absence of systemic and metabolic disease other than obesity and T2DM; 2) absence of malignancy, infection, hepatic diseases, renal diseases, neurological diseases, cardiovascular events and endocrine dysfunction; and 3) absence of history of drug or alcohol abuse, defined as >80 g/day in men and >40 g/day in women. Recruited subjects are tagged in **Figure 1**. All subjects were informed of the purpose of the study and signed the consent. This study was approved by the Ethics Committee of the Second Hospital of Jilin University. Plasma of these subjects was withdrawn and stored at −80°C until analysis.

### 2.2 Genetic testing and data analysis

Whole-genome re-sequencing was performed in the HiSeq X ten PE150 NovaSeq 6000 system in Shanghai Genechem Company Limited on genomic DNA to identify copy number variations (CNVs), single nucleotide polymorphisms (SNPs), insertion-deletion (InDels), and chromosomal structural variations (SVs) in eight subjects from this pedigree. All data were processed using FastQC software developed by Babraham Bioinformatics. Sequence alignment was performed using Burrows-Wheeler Aligner (BWA) software (31). HaplotypeCaller software was applied for mutation detection (32). Variants were identified through SNV calling, SV calling and SNP calling. Annovar software was used to annotate any detected mutations (19).

### 2.3 Gene function enrichment analysis

Genes affected by CNVs, SVs, SNPs or InDels were selected for annotation by comparing with the reference genome. Genes or the corresponding proteins were uploaded to FunRich (Version 3.1.4) for GO classification, including cell component (CC), biological process (BP), molecular function (MF) and biological pathway enrichment analysis. The go-CC, GO-BP, GO-MF and biological pathway with *P* < 0.05 were identified as significant. Meanwhile, proteins were mapped using the online Search Tool for the Retrieval of Interacting Genes (STRING) database (https://string-db.org/version11.5) to construct the PPI network and identify possible relationships between proteins. The PPI network was constructed by setting the minimum required interaction score to medium confidence (0.4). The active interaction sources included were “texming”, “experiments”, “database”, “co-expressing”, “neighborhood”, “genefusion” and “co-occurence”.

### 2.4 Cell culture and treatment

Human bronchial epithelial cells(BEAS-2B) were from National Collection of Authenticated Cell Cultures (Shanghai, China). The cells were cultured in RPMI 1640 medium (ThermoFisher Scientific, USA) supplemented with 2 mM L-glutamine, 100 U/mL penicillin, 100 µg/mL streptomycin, and 10% fetal bovine serum (FBS; Hyclone Laboratories, USA). The cells were grown at 37°C in 5% CO_2_ fully humidified air and were subcultured twice weekly. The cells were seeded in wells of a 6-well plate at 1×10^5^ cells/well. When growth was confluent, the cells were incubated in RPMI 1640 medium containing certain concentrations of D-Glucose (Sigma-Aldrich, USA) for the indicated times. The cell proliferation and viability of BEAS-2B cells was quantified by CCK-8 Kit (Beyotime Biotechnology, China).

### 2.5 Cell viability assays

BEAS-2B cells were seeded into a 96-well plate at 1x10^4^ cells/well with 100 µl of 10% FBS RPMI1640 medium. After overnight incubation, the complete medium containing different concentrations of glucose (15 mM, 20 mM, 25 mM, 30 mM) replaced the original medium of each group for 12h, 24h, 48h and 72h. Then, 10 µl of CCK-8 solution was added to the medium of each group. After the cells were incubated in the dark at 37°C for an additional 1 h, the absorbance at a 450nm wavelength was detected. Then cell viability of each group was calculated.

### 2.6 RT-PCR analysis of MUC5B mRNA

Isolation of total RNA from the cultured cells was performed according to the manufacturer’s instructions of cell total RNA isolation kit (ForeGene, China). Each sample was reverse transcribed into cDNA using the Prime Script RT Regent Kit (Takara, Japan). The primer sequences used in the PCR were 5´- GCCCACATCTCCACC TATGAT-3´ (sense) and 5´-GCAGTTCTCGTTGTCCGTCA-3´ (antisense) for *MUC5B*. Real-time PCR was performed with the SYBR Green Realtime PCR Master Mix Kit (Solarbio, China). Data were normalized versus *GAPDH*. According to the *Ct* values, the expression of *MUC5B* relative to *GAPDH* was calculated by using 2^-ΔΔCt^ formula.

### 2.7 Enzyme-linked immunosorbent assay

The protein levels of MUC5B, IL-1β and IL-6 were determined by enzyme-linked immunosorbent assay (ELISA). The standard substance and samples of cell supernatants were prepared, and incubated at 37°C in the 96-well plate for 2 hours. The standard substance and samples were discarded, and then the plate was blocked with biotin-labeled antibody for 1 hour at 37°C. Wells were then washed three times with the washing buffer, and horseradish peroxidase(HRP)-conjugated secondary antibody was added to wells. 1 hour later at 37°C, wells were washed three times with the washing buffer, and the substrate solution was added to wells, followed by the incubation at 37°C for 20min from light. Color was developed using stopping solution. Optical densities were measured using an ELISA reader (BioTek Instruments, USA) at 450 nm and 570nm. According to the standard curve, the corresponding concentration of each sample was calculated.

### 2.8 Western blot

Human BEAS-2B bronchial epithelial cells were seeded in a 6-well plate and treated with glucose for the indicated times and concentrations. The cells were then washed with cold PBS, and then exposed to the cold lysis buffer (50 mM Tris-HCl, pH 8.0, 5 mM EDTA, 150 mM NaCl, 1% Triton X-100, 1 mM phenylmethylsulfonyl fluoride, protease inhibitor cocktail, and bromophenol blue). The proteins were separated using sodium dodecyl sulfate-polyacrylamide gel electrophoresis and electroblotted onto a nitrocellulose membrane. The memebrane was then blocked with 5% nonfat dry milk in 25 mM Tris-HCl, 150 mM NaCl, and 0.2% Tween-20, and then incubated with the indicated primary antibody of p-JNK (#4668, Cell Signaling Technologies, USA), p-ERK1/2 (#4370, Cell Signaling Technologies, USA), ERK1/2 (#4695, Cell Signaling Technologies, USA), p-p38 (#4511, Cell Signaling Technologies, USA), p-IκBα (#2859, Cell Signaling Technologies, USA), and β-actin (#4970, Cell Signaling Technologies, USA), JNK (#9252, Cell Signaling Technologies, USA), P38 (#9212, Cell Signaling Technologies, USA), IκBα (#4812, Cell Signaling Technologies, USA) overnight at 4°C. Subsequently, the membrane was washed and incubated for 2 hour with secondary antibody conjugated to HRP (#7074,Cell Signaling Technologies, USA). Finally, the membrane was developed using an chemiluminescence reagent kit (Bio-Rad, USA) and exposed to the imager.

### 2.9 Cell transfection with shRNA for MUC5B

The *MUC5B* shRNA knockout plasmid (Psuper-MUC5B-SH) and the negative control shRNA-NC were designed and synthesized by Jiangsu Cencefe Co., Ltd. ShRNA sequence was designed according to the target gene sequence (5’-GGGAAGTCATCT ACAATAAGACC-3’) as follows: Top-Bgl II(60bp) 5’-gatccccGAAGTCATCTACAATAAGATTCAAGAGATCTTATTGTAGATGACTTCTTTTTa-3’; Bottom-Xho I(60bp), 5’-tcgat AAAAAGAAGTCATCTACAATAAGATCTCTTGAATCTTATTGTAGATGACTTCggg-3’.Plasmid transfection was carried out using the transfection reagent Lipofectamine 2000 (Invitrogen, USA), and the procedure was as follows according to the kit instruction (Invitrogen). Briefly, BEAS-2B cells were seeded in wells of a 6-well plate at 2×10 ^5^ cells/well and incubated in RPMI 1640 medium. When the cells were confluent to 80%, MUC5B shRNA and Lipofectamine 2000 were incubated together in RPMI 1640 medium without serum to form a MUC5B shRNA-Lipofectamine complex. After the cells were washed with PBS, the complex-containing medium was then added to each well. After 48 hours of transfection with MUC5B shRNA, the cells were harvested for RT-PCR analysis of MUC5B mRNA. The same procedure was performed with control shRNA.

### 2.10 Statistical analysis

The results were expressed by mean ± standard deviation, analyzed and plotted by GraphPad Prism 6. The data were normally distributed by Pearson test. Comparisons were made using the Student *t*-test between two groups, one-way ANOVA test between multiple groups. Student-Newman-Keuls *post hoc* test was applied. For all tests, *P*-value less than 0.05 was considered statistically significant.

## 3 Results

### 3.1 Clinical data

**Figure 2:**
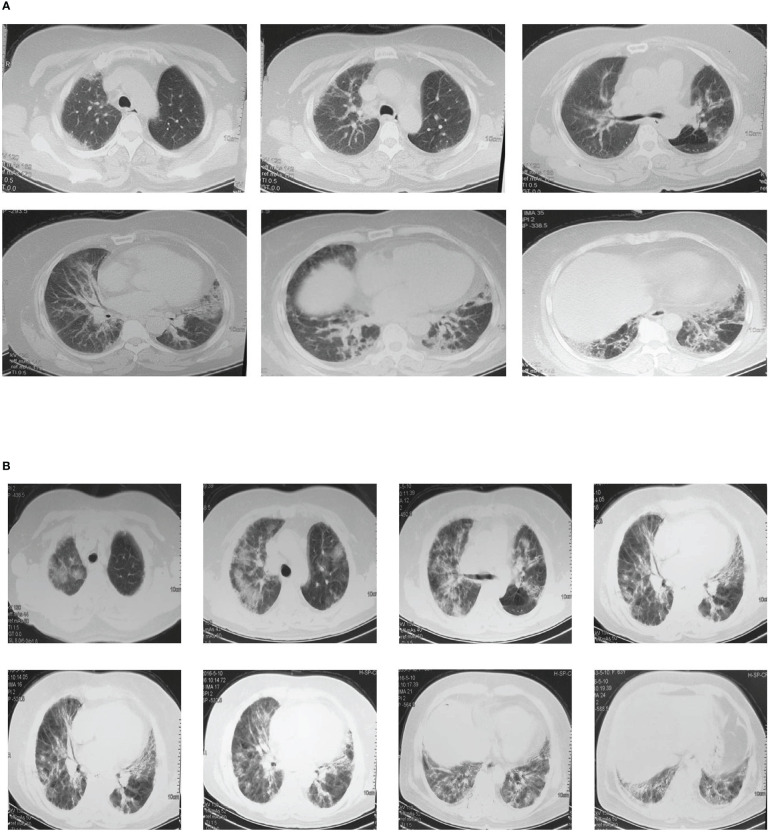
Chest CT images of the proband. **(A)** Multiple ground-glass and grid shadows distributing around the lungs and under the pleura were found in both lungs In December 2015. **(B)** Multiple patchy shadows, grid shadows and cable shadows were diffusely distributed in both lungs, and some of the shadows fused into large areas in May 2016.

The introduction of this pedigree:

NO.203, the proband of the pedigree, female, 61 years old, homemaker. In December 2015, the patient was first admitted to the outpatient department of our hospital for dyspnea, and diagnosed as “ILD” (**Figure 2A**) and “T2DM”. However, the patient did not follow the doctor’s advice for treatment. Since then, her dyspnea had been progressively aggravated. In May 2016, the patient was admitted to the inpatient department for severe dyspnea. Past medical history: coronary heart disease for 20 years, T2DM for 30 years. She denied the history of long-term smoking, and the exposure to special drugs, dust and poison. Her deceased mother also suffered from T2DM and ILD. Physical examination on admission: tachypnea, cyanosis, right jugular vein swelling, crackles rale (Velcro) in both lower lungs by auscultation. Arterial blood gas analysis: pH 7.35, PCO2 35mmHg, PO2 57mmHg, SaO2 86%. Sinus tachycardia, pulmonary P wave and right ventricular high voltage could be detected in her electrocardiogram. Right ventricular hypertrophy, severe pulmonary hypertension, and left ventricular ejection fraction 70% were shown in her color doppler echocardiography. Chest CT images were shown in **Figure 2B**. Laboratory tests: WBC 11.0x10^9^/L, neutrophil percentage 80%, hemoglobin 170g/L, platelet was normal in the blood routine test; the urine routine, liver and kidney function tests, D-dimer, procalcitonin, and fungal-D-glucan were all at normal level; brain natriuretic peptide (BNP) 126pg/mL; the antinuclear antibody spectrum, anti-neutrophil cytoplasmic antibodies, serum complements, rheumatoid factor, cyclic citrullinated peptide, immuno-globulins and anti-cardiolipin antibody were all negative or normal. Besides, no abnormalities were found in the tumor markers. The patient was given antibiotics and intravenous glucocorticoid, however, her condition improved slightly. Unfortunately, the patient died at home in late 2016.

NO.101, who had been deceased when the pedigree was investigated, suffered from T2DM and ILD.

NO.201, who had been deceased at the age of 38, suffered from T2DM and ILD.

NO.202, who had been deceased at the age of 40 when the pedigree was investigated, suffered from T2DM and ILD.

NO.204, who was still alive when the pedigree was investigated, had a history of T2DM for 8 years and ILD for 2 years. The patients once received more than 1 year of oral glucocorticoid treatment, which had been stopped when the pedigree was investigated. The insulin was still regularly used to treat her diabetes.

NO.205, who had been deceased at the age of 53 when the pedigree was investigated, suffered from T2DM and ILD.

NO.206, who had a history of T2DM for 5 years and denied any other diseases, was receiving insulin treatment when the pedigree was investigated. No abnormal changes showed in her chest HRCT images.

NO.303, who had a history of T2DM for 5 years, ILD for 1 year, was receiving regular oral glucocorticoid and insulin treatment when the pedigree was investigated.

NO.304, who had a history of T2DM for 6 years and denied any other diseases, was receiving regular insulin treatment when the pedigree was investigated. No abnormal changes showed in his chest HRCT images.

NO.307, 308, 301, 302, 311, 305, 306, 309 and 310: They didn’t have diabetes, cough, dyspnea and other respiratory symptoms. No abnormal changes showed in their chest HRCT images.

According to the inclusion criteria and informed consent, NO.204 (the proband’s younger sister), NO.206 (the proband’s youngest sister), NO.303 (the son of the proband’s eldest sister), NO.304 (the eldest son of the proband’s eldest brother), NO.305 (the second son of the proband’s eldest brother), NO.307 (son of the proband), NO.308 (daughter of the proband) and NO.309 (the son of the proband’s younger sister) of the pedigree map were included in this study. DNA was extracted from the blood of each subject, and the eight samples were re-sequenced to obtain the data of CNVs, SVs, SNPs and InDels from each sample.

### 3.2 Genetic variation data

#### 3.2.1 CNV findings

We compared the sequencing data of each DNA sample with that of a control sample, and detected CNVs through the different distribution of reads. In this study, 23 genes existing in at least 7 subjects (> 80%) that might be affected by CNVs were selected out as shown in **Table 1**.

**Table 1:** Summary of common genes affected by CNVs.

CNV Region	Type	Gene
chr2:109745796-110262413	Gain	*SH3RF3*
chr4:69512114-69536694	Gain	*UGT2B15*
chr6:1582595-1593195	Gain	*TRIM31*
chr6:2747876-2764695	Gain	*MICB*
chr6:1485581-1498602	Gain	*TRIM40*
chr6:3521493-3600757	Gain	*C6orf10*
chr1:249200241-249213545	Loss	*PGBD2*
chr10:35415568-35502086	Loss	*CREM*
chr13:115079764-115093003	Loss	*CHAMP1*
chr16:70147328-70195384	Loss	*PDPR*
chr16:81115351-81130180	Loss	*GCSH*
chr19:58595008-58629993	Loss	*ZSCAN18*
chr2:171626991-171655681	Loss	*ERICH2*
chr20:1544828-1600889	Loss	*SIRPB1*
chr22:39378203-39388984	Loss	*APOBEC3B*
chr22:51195313-51238265	Loss	*RPL23AP82*
chr3:50387925-50405828	Loss	*CYB561D2*
chr3:96533224-97467986	Loss	*EPHA6*
chr3:123813357-124440236	Loss	*KALRN*
chr3:183967244-184011019	Loss	*ECE2*
chr6:1550103-1642179	Loss	*HCG17*
chr6:1603064-1643201	Loss	*HCG18*
chr9:139221731-139254257	Loss	*GPSM1*

GO analysis and biological pathway analysis about the 23 genes were carried out. About cellular components, genes related to the nucleus accounted for the largest proportion. The molecular functions of 21.1% of the genes were unknown, while the rest genes were related to transcription factor activity, receptor activity, transferase activity and DNA binding. About biological processes, genes related to the metabolism of base, nucleoside, nucleotide and nucleic acid were the most abundant, followed by genes related to signal transduction and cell communication. The 23 genes were involved in many signaling pathways, such as ARF6 signaling pathway, PI3K signaling pathway, mTOR signaling pathway, ErbB signaling pathway, S1P1 signaling pathway, etc. Further analysis in STRING database didn’t detect significant enrichment.

According to the literature, the relevant studies on the 17 genes affected by CNV, including *SH3RF3, UGT2B15, TRIM31, MICB, TRIM40, C6orf10, PGBD2, CHAMP1, PDPR, ZSCAN18, ERICH2, SIRPB1, APOBEC3B, RPL23AP82, CYB561D2, EPHA6* and *HCG17*, were not found in T2DM or ILD. The other 6 genes, including *CREM, GCSH, KALRN, ECE2, HCG18* and *GPSM*1, had a certain role in the pathogenesis of T2DM and its complications. However, their roles in the development of ILD had not been studied yet.

#### 3.2.2 SV findings

SVs were detected by comparing the sequencing data of each sample with that of a control sample. In this study, 190 genes existing in at least 7 subjects (> 80%) that might be affected by SVs were selected out as shown in **Table 2**.

**Table 2:** Summary of common genes affected by SVs.

chrA	posA	ortA	chrB	posB	ortB	Type	GeneNameA	GeneNameB
chr1	30878819	–	chr1	30878513	+	ITX	LOC101929406,MATN1	LOC101929406,MATN1
chr1	53595128	+	chr1	53595604	+	DEL	SLC1A7	SLC1A7
chr1	96945545	+	chr3	114915084	–	CTX	LOC101928241,PTBP2	ZBTB20,GAP43
chr1	145092948	+	chr1	145097082	+	DEL	NBPF20,NBPF9	NBPF20,NBPF9,SEC22B
chr1	187466730	–	chr1	187466476	+	ITX	LINC01036,NONE	LINC01036,NONE
chr2	37453432	+	chr19	29855782	–	CTX	CEBPZ	LOC284395
chr2	41973156	+	chr2	41975866	+	DEL	SLC8A1,LOC388942	SLC8A1,LOC388942
chr2	119653329	+	chr2	119659369	+	DEL	EN1,MARCO	EN1,MARCO
chr2	123364878	+	chr2	123365377	+	DEL	TSN,CNTNAP5	TSN,CNTNAP5
chr2	138245304	+	chr2	138245633	+	DEL	THSD7B	THSD7B
chr2	173616807	+	chr2	173616766	+	INS	RAPGEF4	RAPGEF4
chr2	191002633	+	chr2	191002548	+	INS	C2orf88	C2orf88
chr2	236818929	+	chr2	236818861	+	INS	AGAP1	AGAP1
chr3	12696232	+	chr10	101851839	+	CTX	RAF1	CPN1,ERLIN1
chr3	31881392	+	chr14	29261491	+	CTX	OSBPL10	LINC01551
chr3	99940769	+	chr1	79582083	+	CTX	TMEM30C,TBC1D23	ELTD1,LOC101927412
chr3	100868474	+	chr3	100868430	+	INS	ABI3BP,IMPG2	ABI3BP,IMPG2
chr3	144693111	+	chr3	144693237	+	DEL	C3orf58,PLOD2	C3orf58,PLOD2
chr4	53155166	+	chr4	53155079	+	INS	SPATA18,USP46	SPATA18,USP46
chr4	78199225	+	chr4	78199274	+	DEL	CCNG2,CXCL13	CCNG2,CXCL13
chr4	88858700	–	chr4	88847164	+	ITX	MEPE,SPP1	MEPE,SPP1
chr4	162776134	+	chr4	162776213	+	DEL	FSTL5	FSTL5
chr4	187386699	+	chr4	187386636	+	INS	F11-AS1	F11-AS1
chr4	86442606	+	chr4	86442559	+	INS	ARHGAP24	ARHGAP24
chr4	157581838	+	chr4	157581700	+	INS	CTSO,PDGFC	CTSO,PDGFC
chr4	80894475	+	chr5	21207714	+	CTX	ANTXR2	CDH18,GUSBP1
chr5	152272036	+	chr14	52667761	+	CTX	LINC01470	NID2,PTGDR
chr5	28934877	–	chr5	28932856	+	ITX	LSP1P3,LOC101929645	LSP1P3,LOC101929645
chr5	91480787	+	chr5	91481129	+	DEL	ARRDC3-AS1,NR2F1-AS1	ARRDC3-AS1,NR2F1-AS1
chr5	114739725	–	chr8	117398602	+	CTX	CCDC112,FEM1C	LINC00536,EIF3H
chr5	143413889	+	chr10	127633806	+	CTX	HMHB1,YIPF5	FANK1
chr5	143512867	+	chr5	143515048	+	DEL	HMHB1,YIPF5	HMHB1,YIPF5
chr6	6034770	+	chr5	7411901	+	CTX	NRN1,F13A1	ADCY2
chr6	8902225	+	chr6	8902168	+	INS	LOC100506207,TFAP2A	LOC100506207,TFAP2A
chr6	43655533	+	chr9	33130549	+	CTX	MRPS18A	B4GALT1
chr6	44264732	+	chr6	44264682	+	INS	TCTE1	TCTE1
chr6	57284911	+	chr6	57289357	+	DEL	PRIM2	PRIM2
chr6	120764923	+	chr6	120764817	+	INS	LOC285762,TBC1D32	LOC285762,TBC1D32
chr6	136582615	+	chr6	136589300	+	DEL	BCLAF1	BCLAF1
chr6	151777322	+	chr19	29855783	+	CTX	C6orf211	LOC284395
chr7	22853488	+	chr18	38517914	+	CTX	TOMM7	LINC01477,KC6
chr7	67120984	+	chr7	67121048	+	DEL	LINC01372,LOC102723427	LINC01372,LOC102723427
chr7	113416176	+	chr7	113422208	+	DEL	LINC00998,PPP1R3A	LINC00998,PPP1R3A
chr7	158180267	+	chr7	158180207	+	INS	PTPRN2	PTPRN2
chr8	23407223	+	chr8	23407860	+	DEL	SLC25A37	SLC25A37
chr8	24779109	+	chr13	101172615	+	CTX	NEFM,NEFL	PCCA
chr8	25327221	+	chr8	25327279	+	DEL	CDCA2	CDCA2
chr8	41270459	–	chr8	67615798	+	ITX	SFRP1,GOLGA7	C8orf44-SGK3
chr8	70839998	+	chr8	70840048	+	DEL	SLCO5A1,PRDM14	SLCO5A1,PRDM14
chr8	74044179	+	chr8	74044096	+	INS	SBSPON,C8orf89	SBSPON,C8orf89
chr8	92153187	+	chr11	104786600	–	CTX	LRRC69	LOC643733
chr8	107207882	–	chr1	11096278	+	CTX	ZFPM2-AS1,OXR1	MASP2
chr8	108569293	+	chr8	108569363	–	ITX	ANGPT1,RSPO2	ANGPT1,RSPO2
chr9	9413418	+	chr13	90947648	+	CTX	PTPRD	MIR622,LINC01049
chr9	88661978	+	chr9	88661918	+	INS	GOLM1	GOLM1
chr9	140772669	+	chr9	140773505	+	DEL	CACNA1B	CACNA1B
chr10	8717568	+	chr10	8717528	+	INS	LINC00708,LOC101928272	LINC00708,LOC101928272
chr10	48419946	–	chr5	100389387	+	CTX	GDF2,GDF10	ST8SIA4,SLCO4C1
chr10	54941242	+	chr10	54941198	+	INS	MBL2,PCDH15	MBL2,PCDH15
chr10	60984921	+	chr10	9972888	+	INS	PHYHIPL	LOC101928272,LOC101928298
chr10	67032386	+	chr10	67032802	+	DEL	ANXA2P3,LINC01515	ANXA2P3,LINC01515
chr10	74842608	+	chr10	74842833	+	DEL	P4HA1	P4HA1
chr12	50973716	+	chr12	50975506	+	DEL	DIP2B	DIP2B
chr12	74014507	+	chr7	125264207	+	CTX	LOC101928137,LOC100507377	LOC101928283,GRM8
chr13	87392949	–	chr7	44495374	+	CTX	SLITRK6,MIR4500HG	NUDCD3
chr13	73169686	+	chr7	127929321	+	CTX	DACH1,MZT1	LEP,MGC27345
chr13	111076796	–	chr13	41271120	+	ITX	COL4A2	FOXO1,MIR320D1
chr14	58871151	+	chr7	82430515	+	CTX	TOMM20L	PCLO
chr14	106484225	+	chr15	22486821	+	CTX	ADAM6,LINC00226	OR4N3P,REREP3
chr15	40854180	–	chr7	26241365	+	CTX	C15orf57	CBX3
chr15	62947784	+	chr15	62947921	+	DEL	TLN2	TLN2
chr15	91214612	+	chr19	29956114	+	CTX	LOC101926895	LOC284395
chr16	8739884	+	chr16	8739844	+	INS	METTL22	METTL22
chr16	22908973	+	chr16	22909252	+	DEL	HS3ST2	HS3ST2
chr16	54454410	+	chr4	42088050	–	CTX	IRX3,CRNDE	SLC30A9
chr17	29729944	+	chr12	46175163	+	CTX	RAB11FIP4	ARID2
chr17	30893853	–	chr6	161181867	+	CTX	MYO1D	PLG,MAP3K4
chr17	68624947	+	chr20	8670606	+	CTX	KCNJ2,CASC17	PLCB1
chr17	10886858	+	chr17	10895732	+	DEL	PIRT,SHISA6	PIRT,SHISA6
chr18	11572230	+	chr18	11572179	+	INS	LINC01255,SLC35G4	LINC01255,SLC35G4
chr18	25871823	+	chr20	32335134	+	CTX	CDH2,MIR302F	ZNF341
chr18	35306060	+	chr18	35306633	+	DEL	MIR4318,LINC00669	MIR4318,LINC00669
chr18	54706185	+	chr8	96730502	+	CTX	WDR7,LINC-ROR	C8orf37-AS1
chr18	66572496	–	chr6	76031227	+	CTX	CCDC102B	FILIP1
chr18	75266998	+	chr18	75268160	+	DEL	GALR1,LINC01029	GALR1,LINC01029
chr19	46175262	–	chr6	24384210	+	CTX	GIPR	DCDC2
chr20	6302495	+	chr7	107829261	–	CTX	FERMT1,CASC20	NRCAM
chr20	42300915	+	chr20	42300870	+	INS	MYBL2	MYBL2
chr20	53292970	+	chr2	115390731	–	CTX	DOK5,LINC01441	DPP10
chr20	61661208	+	chr20	61661143	+	INS	LOC63930	LOC63930
chr20	62709634	+	chr20	62709531	+	INS	RGS19	RGS19
chr20	6302495	+	chr7	107829261	–	CTX	FERMT1,CASC20	NRCAM

ChrA= the chromosome on one side of SV; PosA=the position on one side of SV; ortA= the plus or minus strand of this position ChrB= the chromosome on the other side of SV; PosB= the position on the other side of SV; ortB= the plus or minus strand of this position Type= Deletion (DEL),Insertion (INS), Inversion(INV), Intrachromosomal translocation (ITX), Interchromosomal translocation (CTX); GeneNameA= the name of the gene on one side of SV GeneNameB= the name of the gene on the other side of SV.

**Figure 3:**
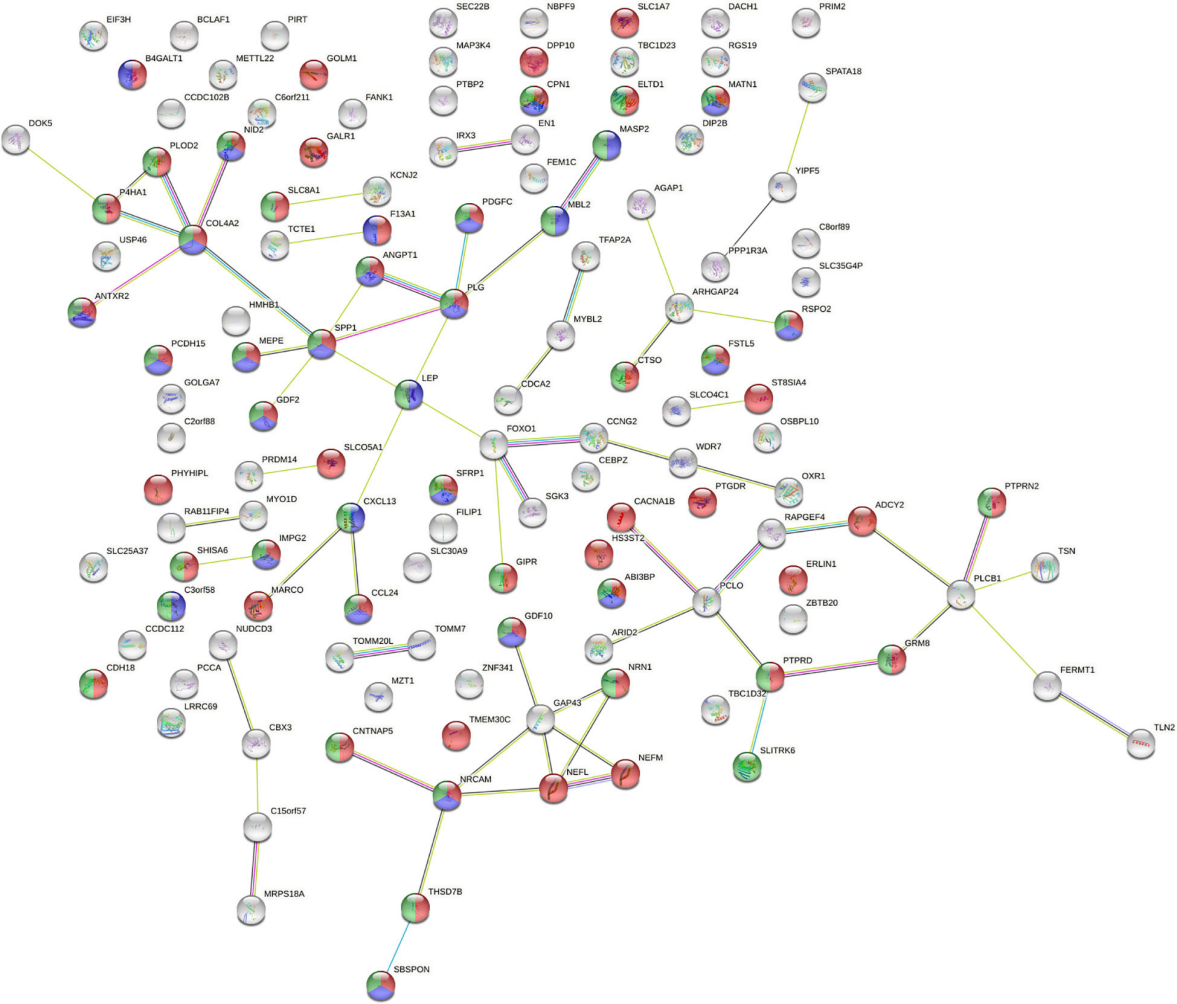
Analysis of protein-protein interaction network (PPI) affected by SVs. The number of nodes was 130, with each node representing a protein, 53 red nodes representing glycoproteins, 41 green nodes representing cell signal transduction, and 28 blue nodes representing secretory proteins. There were a total of 68 lines between nodes, each line representing the interaction between two proteins. Red line – gene fusion, green line – gene neighborhood, blue line – gene co-occurrence, purple line – experimentally determined, yellow line – textming, light blue line – from curated databases, black line – co-expression. PPI enrichment *P* = 1.57x10^-4^.

The 190 genes existing in at least 7 subjects which were affected by SVs all distributed on autosomes. About cell components, genes related to extracellular components accounted for the highest proportion. About cell function, the percentages of genes related to cell adhesion molecule activity, extracellular matrix composition and growth factor activity ranked among the top three. According to the biological process, the proportion of genes related to cell signal transduction and cell communication ranked among the top two. By biological pathway analysis, we found that the main pathways in which these genes were involved included neuronal system biological processes, EMT, chemical synaptic and postsynaptic transmission signaling. Further, the PPI network (**Figure 3**) of the proteins corresponding to the genes affected by SVs was constructed in the STRING database. The PPI network revealed that 15 proteins were associated with extracellular matrix components, 42 were involved in cell signal transduction, 54 belonged to glycoproteins, and 28 belonged to secretory proteins. The hub nodes and the four-color nodes in the PPI (the four-color nodes represented the proteins belonging to secretory protein, glycoprotein and extracellular matrix component at the same time, and also participating signal transduction) were analyzed, including PLG, MATN1, ANGPT1, MEPE, COL4A2, NID2, IMPG2, GDF10, SFRP1, ABI3BP, SBSPON, CDH2, SPP1, PTPRD and NRCAM. Whether each node played a role in the development of T2DM or ILD was still unclear. In addition, whether the genes corresponding to these nodes would form fusion genes, affect gene expression or change their phenotypes after being affected by SV had not been studied yet.

#### 3.2.3 SNPs and InDels findings

20263-20507 SNPs in 3’-UTR, 5’-UTR and exonic regions were genotyped in each subject, including synonymous, non-synonymous, stop-gain and stop-loss. 510-587 InDels were genotyped in each subject, including non-frameshift deletion, non- frameshift insertion, frameshift deletion, frameshift insertion, stop-gain, and stop-loss.

70 common InDels distributed in the coding region on the autosomes and X chromosomes in at least 7 subjects, including 40 frameshift deletions and frameshift insertions. The genes with frameshift deletions are shown in **Table 3**, and the genes with frameshift insertions are shown in **Table 4**.

**Table 3:** The common genes with frameshift deletions.

Chr	Region	Deleted sequence	Gene name
chr1	1q44	A	*OR2B11*
chr1	1q44	CAGCACG	*OR2T35*
chr1	1p36.11	CC	*UBXN11*
chr3	3p25.1	C	*SLC6A6*
chr3	3p12.3	C	*ZNF717*
chr4	4q31.3	TTTG	*DCHS2*
chr4	4q31.1	C	*MAML3*
chr4	4q31.1	GCTGCTGCTGC	*MAML3*
chr5	5q32	T	*TIGD6*
chr6	6q25.3	TGGTAAGT	*SLC22A1*
chr6	6q27	GC	*TBP*
chr7	7q11.23	A	*POMZP3*
chr8	8q24.3	GGGGGTGCAAGGTGA	*ADCK5*
chr8	8p23.1	AAC	*ERI1*
chr8	8p21.3	C	*NUDT18*
chr9	9p11.2	G	*CNTNAP3B*
chr9	9q31.1	GC	*OR13C2*
chr9	9q31.1	GTTA	*OR13C2*
chr9	9q31.1	T	*OR13C5*
chr11	11q12.2	TT	*MS4A14*
chr11	11p15.4	G	*OR52B4*
chr12	12q13.3	AT	*PTGES3*
chr12	12q24.31	CCGCCA	*ORAI1*
chr14	14q32.33	GACGGGCAG	*C14orf180*
chr15	15q13.2	CA	*CHRFAM7A*
chr15	15q11.2	T	*GOLGA6L2*
chr16	16q24.3	GGTGTG	*CTU2*
chr16	16q23.2	TT	*PKD1L2*
chr16	16q24.2	GA	*ZFPM1*
chr17	17q21.2	A	*KRT24*
chr17	17p13.2	G	*P2RX5*
chr17	17q11.2	A	*SARM1*
chr19	19p13.3	AGCTGGCCGGGGAGG	*HDGFRP2*
chr19	19q13.41	TG	*ZNF480*
chr21	21q22.3	GGCCCCCCA	*COL18A1*
chr21	21q22.3	TC	*KRTAP10-1*
chr21	21q22.11	G	*KRTAP19-6*
chr21	21q22.11	A	*SON*
chrX	Xp11.22	TCCTCGAGGCAGCC	*NUDT11*
chrX	Xq22.3	A	*TEX13A*

**Table 4:** The common genes with frameshift insertions.

Chr	Region	Inserted sequence	Gene name
chr1	1q23.1	AC	*GPATCH4*
chr2	2q37.1	CG	*GIGYF2*
chr3	3q13.2	A	*ATG3*
chr5	5q31.3	GT	*SRA1*
chr5	5q33.3	C	*CYFIP2*
chr7	7q11.23	C	*SRRM3*
chr7	7q36.1	C	*SSPO*
chr9	9q33.2	A	*OR1B1*
chr10	10q25.3	TT	*ATRNL1*
chr11	11p15.4	AC	*C11orf40*
chr12	12p13.33	C	*WNK1*
chr12	12q13.11	A	*OR10AD1*
chr12	12p13.31	TAAGT	*CLECL1*
chr13	13q22.3	GG	*SLAIN1*
chr14	14q32.12	G	*ATXN3*
chr16	16q24.3	GTGA	*ZNF778*
chr17	17p13.1	G	*C17orf100*
chr17	17q11.2	G	*SARM1*
chr17	17q25.1	T	*LOC100134391*
chr17	17q25.1	T	*LOC100134391*
chr19	19q13.41	G	*VSIG10L*
chr19	19q13.42	C	*SBK3*
chr20	20p13	GCCCC	*GNRH2*
chr21	21q22.11	A	*SON*
chr21	21q22.3	G	*PRDM15*
chr21	21q22.3	TG	*KRTAP10-1*
chr22	22q11.21	C	*CLTCL1*
chr22	22q11.21	G	*SCARF2*
chrX	Xq22.1	G	*TCEAL6*
chrX	Xq25	G	*GRIA3*

GO classification and biological pathway analysis were performed about the 40 genes with frameshift deletion. About the cell components, the genes related to cell membrane components were in the majority. About the molecular functions, the function of 26.5% of the genes were unknown, while the rest were related to G protein coupled receptor activity and cellular structural molecular activity. In the biological process, the biological process of 26.5% genes was unknown, while the rest were mostly related to cell signaling transduction and cell communication. The signaling pathways in which these genes were involved are numerous, including ARF6 signaling pathway, PI3K signaling pathway, mTOR signaling pathway, ErbB signaling pathway, S1P1 signaling pathway, and IGF1 signaling pathway, etc. Further analysis in STRING database did not detect significant enrichment of the corresponding proteins. According to the literature, only the variants of *SLC22A1, TBP, ORAI1, SARM1* and *COL18A1* were found to play a certain role in the development of T2DM or be the risk factors of T2DM. However, the remaining 35 genes were rarely studied in the field of T2DM or ILD.

GO classification and biological pathway analysis were carried out about the 30 genes with frameshift insertion. About cellular component, genes related to cytoplasm and nucleus accounted for a large proportion. The molecular function of 45.8% of the genes were unknown, while the rest were related to immune protein activity, transcription factor activity, and G-protein-coupled receptor activity. In biological process, the biological process of 33.3% of the genes were unknown, while the remaining related to cell signalling transduction and cell communication accounted for a large proportion. The 30 genes were involved in numerous signaling pathways, such as IL-3-mediated signaling pathway, IL-5-mediated signaling pathway, PEGFR signaling pathway, GMCSF-mediated signaling pathway, ErbB signaling pathway, S1P1 signaling pathway, IGF1 signaling pathway, etc. Further analysis in STRING database did not detect significant enrichment of the corresponding proteins. 5 mutated genes including *GIGYF2, ATG3, SRA1, WNK1* and *CLECL1* were found to be involved in the development of T2DM and its complications, or be the risk factors for T2DM, according to the very few related studies. The remaining 25 genes had not yet been studied in T2DM or ILD.

The number of SNPs detected by each subjects was about 20,000. In order to simplify the search scope and effectively query the SNPs that may be relevant to the T2DM complicated with ILD, we searched according to the mutated genes which had been identified to be associated with the development of ILD (including familial pulmonary fibrosis) in previous studies, including *AKAP13, ATP11A, CDKN1A, DPP9, DSP, ELMOD2, FAM13A, HLA-DRB1, IL1RN, IL8, MAPT, MDGA2, MUC2, MUC5B, OBFC1, SPPL2C, TERC, TERT, TGFB1, TLR3, TOLLIP and TP53 (* 30). 38 SNPs within the specific genes in at least 7 subjects were summarized in **Table 5**.

**Table 5:** The common SNPs within the genes associated with IPF.

Gene	Region	SNP	Alteration	Type
*AKAP13*	3’-UTR 3’-UTR 3’-UTR 3’-UTR 3’-UTR	rs8110 rs13225 rs3169121 rs2542604 rs1808339	A>T C>G T>G C>G A>C	N/A N/A N/A N/A N/A
*ATP11A*	3’-UTR 3’-UTR	rs7985702 rs1046790	T>C T>C	N/A N/A
*DSP*	exonic exonic exonic exonic	rs2806234 rs2076304 rs1016835 rs2744380	T>G G G>A G>C	synonymous synonymous synonymous synonymous
*FAM13A*	5’-UTR	rs2305934	T>C	N/A
*IL1RN*	exonic	rs315952	T>C	synonymous
	3’-UTR	rs315951	C>G	N/A
*MAPT*	exonic	rs2258689	T>C	non-synonymous
*TP53*	5’-UTR	rs2909430	C>T	N/A
*MUC2*	exonic exonic exonic exonic exonic exonic exonic exonic exonic exonic	rs7944723 rs10794292 rs6421972 rs7480563 rs41411848 rs41345745 rs57737240 rs10794288 rs10902088 rs10794291	C>G A>C T>C T>C T>C G>C G>C T>C C>T C>T	synonymous synonymous synonymous synonymous non-synonymous non-synonymous non-synonymous synonymous synonymous synonymous
*MUC5B*	exonic exonic exonic exonic exonic	rs2075859 rs7116614 rs4963031 rs2943531 rs2943512	C>T C>T T>C A>G A>C	synonymous synonymous non-synonymous non-synonymous non-synonymous
*OBFC1*	3’-UTR 3’-UTR exonic exonic 5’-UTR	rs4917405 rs911547 rs10786775 rs2487999 rs4387287	T>C G>A G>C T>C A>C	N/A N/A non-synonymous non-synonymous N/A
*SPPL2C*	exonic exonic	rs242944 rs171443	G>A A>G	synonymous non-synonymous

N/A, Not applicable.

The 38 SNPs were found in 10 genes including *AKAP13, ATP11A, DSP, FAM13A, IL1RN, MAPT, TP53, MUC2, MUC5B, OBFC1* and *SPPL2C*. According to the literature, the roles of *AKAP13* SNPs rs8110, rs13225, rs3169121, rs2542604 and rs1808339, *ATP11A* SNPs rs7985702 and rs1046790, *IL-1RN* SNP RS315951, *TP53* SNP rs2909430, *MUC2* SNPs rs41411848, rs41345745 and rs57737240, *MAPT* SNP rs2258689, *OBFC1* SNPs rs10786775, rs2487999, rs4917405 and rs911547, as well as *SPPL2c* SNPs rs242944 and rs171443 in the pathogenesis of T2DM or ILD were unclear; *OBFC1* SNP rs4387287 might be closely related to the susceptibility of T2DM, but its role in ILD remained unclear; *DSP* rs2076295 and *FAM13A* rs2609255 had been confirmed to be associated with some types of ILD, but their roles in ILD remained unclear; *MUC5B* SNP rs2943512 had been identified to be significantly associated with the susceptibility of T2DM, and the over-expressed MUC5B in the distal airway and alveolar cavity had been confirmed to be closely related to the development of ILD.

To sum up, the roles of most variants in the pathogenesis of T2DM or ILD were unclear. Nevertheless, *MUC5B* SNP rs2943512 (A > C) was considered to be a potentially pathogenic mutation associated with T2DM complicated with ILD. Next, the function experiment of MUC5B in bronchial epithelial cells was carried out, laying a foundation for the mechanism exploration of T2DM complicated with ILD.

### 3.3 The effects of high glucose on the expression of MUC5B in bronchial epithelial cells

#### 3.3.1 High glucose affects viability of BEAS-2B cells

**Figure 4:**
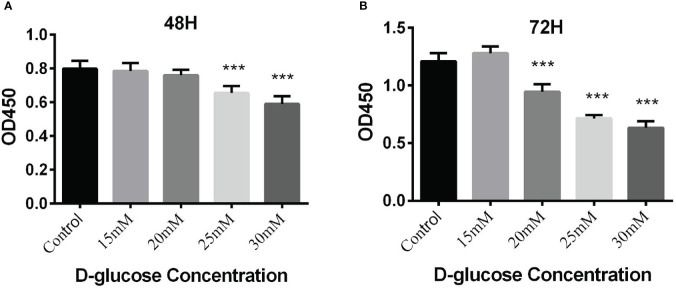
**(A)** Proliferation of BEAS-2B cells cultured in the medium containing different concentrations of glucose for 48 hours by CCK-8 assay; **(B)** Proliferation of BEAS-2B cells cultured in the medium containing different concentrations of glucose for 72 hours by CCK-8 assay (***P < 0.001).

The viability of BEAS-2B cells stimulated by high glucose was detected by CCK-8 assay. The results demonstrated that high glucose 25mM (48h), 30mM (48h), 20mM (72h), 25mM (72h) and 30mM (72h) could inhibited cell growth significantly (***P<0.001) (**Figure 4**).

#### 3.3.2 Effects of high glucose on the expression of MUC5B in BEAS-2B cells

**Figure 5:**
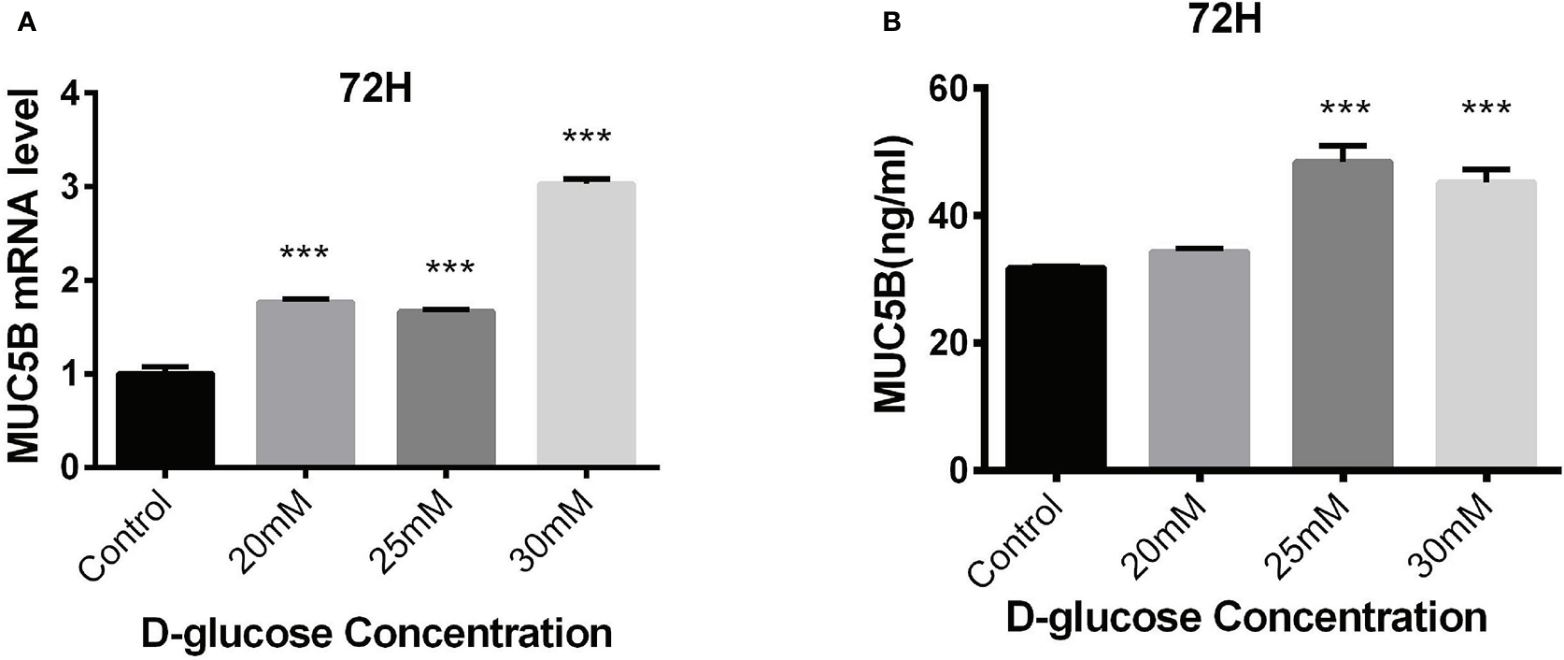
Effects of high glucose on the expression of MUC5B in BEAS-2B cells. **(A)** Compared with the control group, MUC5B mRNA in BEAS-2B cells were significantly increased after 20mM, 25mM and 30mM high-glucose stimulation for 72 hours (***P < 0.001). **(B)** Compared with the control group,MUC5B in the supernatant were significantly increased after 25mM and 30mM high-glucose stimulation for 72 hours (***P < 0.001).

The BEAS-2B cells in normal RPMI1640 medium (D-glucose concentration 11.11mM) was regarded as control, while the cells in medium containing 20mM, 25mM and 30mM glucose for 72h were experimental groups. MUC5B mRNA and MUC5B protein in the supernatant in these groups were detected, respectively. The results showed that compared with the control group, MUC5B mRNA in 20mM, 25mM and 30mM high-glucose groups were statistically increased at 72h (***P < 0.001) (**Figure 5A**), and MUC5B in the supernatant of 25mM and 30mM high-glucose groups were statistically increased at 72h (***P < 0.001) (**Figure 5B**). Finally, 30mM glucose stimulation for 72h was chosen as the subsequent experimental conditions.

#### 3.3.3 Effects of MUC5B on cytokine production in BEAS-2B cells stimulated by high glucose

**Figure 6:**
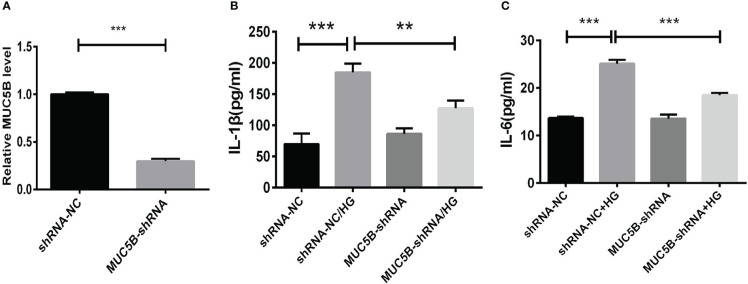
Effects of MUC5B on cytokine production in BEAS-2B cells stimulated by high glucose. **(A)** The efficiency of *MUC5B*-ShRNA transfection into BEAS-2B cells was detected by RT-PCR. After MUC5B knockdown, BEAS-2B cells were stimulated by 30mM glucose for 72 hours. **(B)** IL-1 β and **(C)** IL-6 in the supernatant were both significantly increased compared to the control (**P < 0.01, ***P < 0.001).

To identify the effects of MUC5B on the production of cytokines in BEAS-2B cells, the cells were cultured in medium containing 30mM glucose for 72 h following the transfection of *MUC5B* shRNA into the cells (**Figure 6A**). Subsequently, IL-1 β and IL-6 in the supernatant were detected by ELISA, respectively. (**Figures 6B, C**). The results showed that the concentrate ions of IL-1β and IL-6 in high glucose group were both significantly increased compared to the control (***P < 0.001). While, compared to the high glucose group, the concentrations of IL-1β and IL-6 were both significantly decreased when *MUC5B* was knockdown even stimulated by high glucose (**P <0.01, ***P < 0.001).

#### 3.3.4 Effects of MUC5B on ERK1/2 activation in BEAS-2B cells stimulated by high glucose

**Figure 7:**
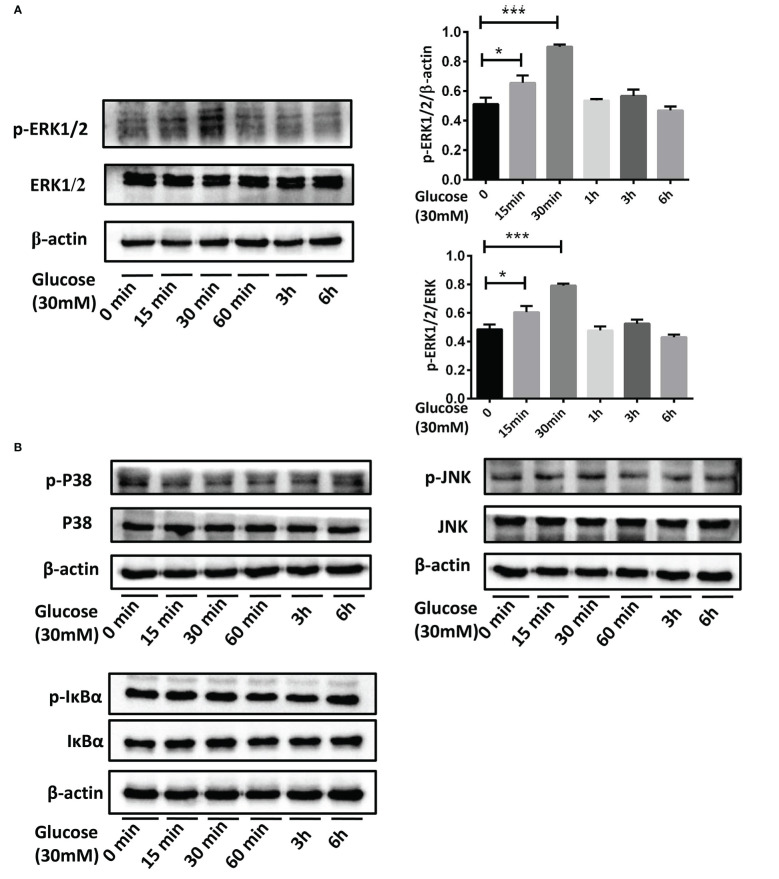
The activation of MAPK and NF-κB pathways in BEAS-2B cells stimulated by high glucose. **(A)** Compared with the control group, p-ERK1/2 in BEAS-2B cells was up-regulated when stimulated by 30mM glucose for 15 minutes and 30 minutes. **(B)** P38, JNK or IκB didn’t activate at each point from 15 minutes to 6 hours when stimulated by 30mM glucose. The blots and densitometry analysis data are representative of three independent experiments. *P <0.05, and ***P < 0.001.

After culturing BEAS-2B cells in the medium containing 30mM glucose for 15min, 30min, 1h, 3h and 6h, p-ERK1/2, p-P38, p-JNK, and p-IκB in each group were detected. The results showed that compared with the control group, p-ERK1/2 was up-regulated when stimulated by the high glucose for 15min and 30min (**Figure 7A**,*P <0.05, ***P < 0.001). However, no change of p-P38, p-JNK or p-IκB was detected at each point from 15min to 6h (**Figure 7B**).

**Figure 8:**
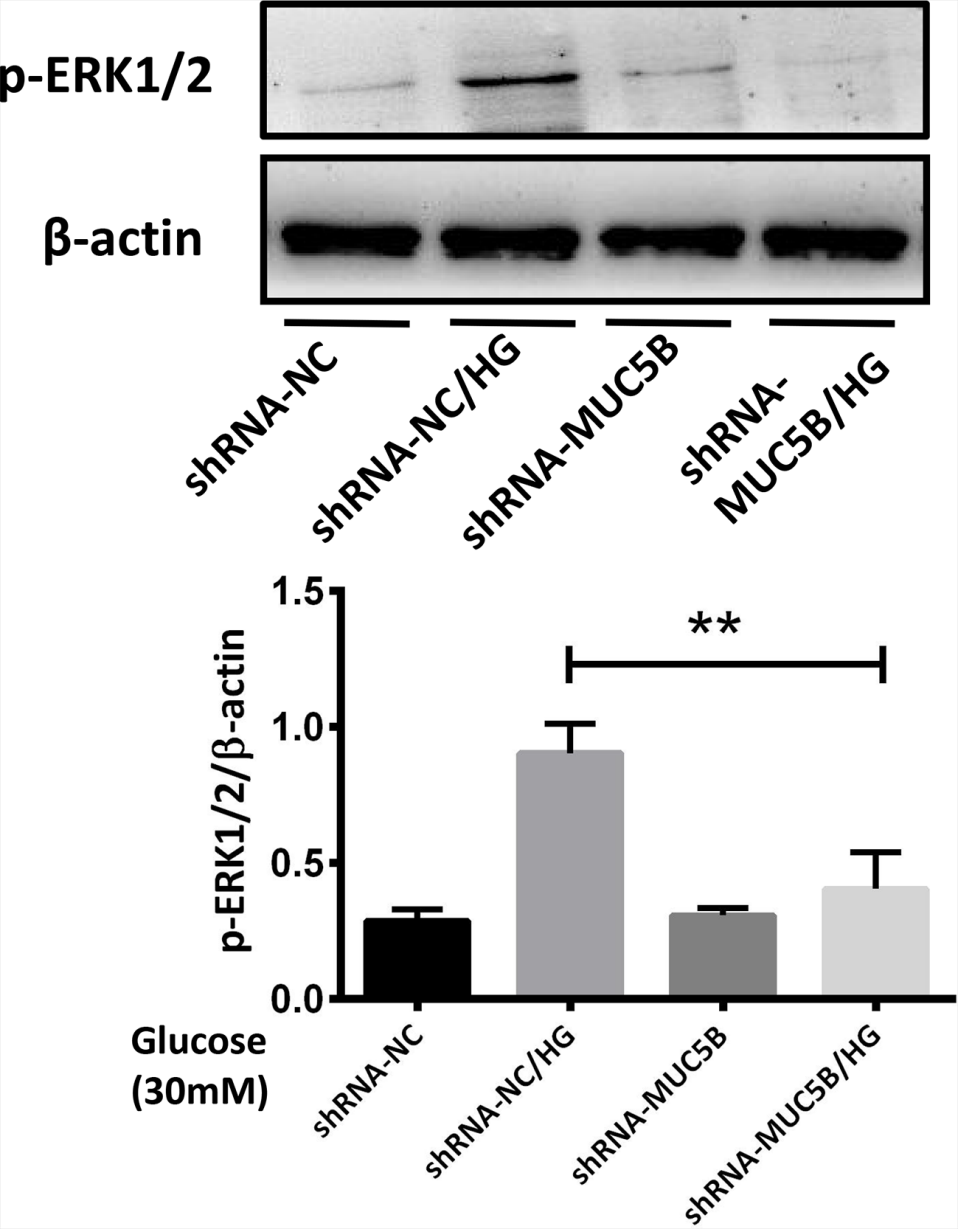
MUC5B promoted ERK1/2 activation in BEAS-2B cells stimulated by high glucose. Compared with high glucose group, p-ERK1/2 in the *MUC5B* knockdown cells stimulated by 30mM glucose for 30min was significantly down-regulated (**P <0.01). The blots and densitometry analysis data are representative of three independent experiments. **P <0.01.

To confirm our suppose that over-expressed MUC5B could promote the synthesis of IL-1β and IL-6 by activating ERK1/2, BEAS-2B cells were stimulated by 30mM glucose for 30min following the silencing of *MUC5B* gene. Subsequently, p-ERK1/2 in each group was detected by western blot. The results showed that compared with the high glucose group, p-ERK1/2 in the *MUC5B* knockdown cells stimulated by high glucose was significantly down-regulated (**Figure 8**, **P <0.01), suggesting over-expressed MUC5B could promote ERK1/2 activation in BEAS-2B cells when stimulated by high glucose.

## 4 Discussion

T2DM and ILD both belong to complex diseases. In recent years, genetic studies about the both diseases have been gradually extensive, and mutations related to the risks or pathogenesis of the two diseases have been constantly revealed. With further studies, it has been recognized that ILD could be a complication of T2DM. However, the relevant studies about genetic variations promoting ILD in T2DM patients are still rare.

In this study, a pedigree with T2DM complicated with ILD including three generations was selected. Familial T2DM is not rare in clinical practice, however, pedigree with both T2DM and ILD are indeed rare. In this pedigree, almost all the first and second generation members had both T2DM and ILD. 8 living members of the pedigree were included as subjects. By conducting the whole-genome re-sequencing of each subject’s blood DNA sample, it was found that the healthy subjects from the third generation also had the same potential pathogenic genetic variants as the patients from the second and third generation. It suggested that T2DM was relevant to the development of ILD, and T2DM complicated with ILD might be heredofamilial.

The common genetic variants of at least 7 subjects were further screened out after gene sequencing, involving numerous SVs, CNVs, SNPs and InDels. Previous studies considered that the susceptibility of many diseases was basically attributed to SNPs, however, SNP can only explain a part of the heritability of diseases. Our study also provided genes which were affected by InDels, SVs and CNVs, so as to provide more genetic information for the mechanism research of ILD in diabetic patients. Up to now, the roles of most variants in T2DM or ILD remained unclear, and only a small part might be involved in the development of T2DM or ILD. While, *MUC5B* variation was the specific one found to be related to both T2DM and ILD in the study, and *MUC5B* SNP rs2943512 (A > C) was considered to be a potentially pathogenic mutation associated with T2DM complicated with ILD. Meanwhile, the over-expressed MUC5B protein in the distal airway and alveolar cavity had also been considered to be closely related to the development of idiopathic pulmonary fibrosis (IPF) (33, 34). Therefore, it was of great significance to investigate the role of MUC5B SNP rs2943512 (A > C) or abnormal MUC5B protein in the development of T2DM complicated with ILD.

*MUC5B* encodes mucin 5B protein, which is a glycosylated macromolecular component of mucus and produced by mucinous cells in bronchial submucosal glands and type II alveolar epithelial (ATII) cells. Normally, MUC5B plays the physiological roles of maintaining airway homeostasis (35), involving capturing inhaled particles and bacteria which are transported out of the airway by cilia oscillating or coughing. In addition, MUC5B could also helps remove endogenous debris, including dead epithelial cells and white blood cells. When MUC5B is over-expressed by stimulus, the capacity of mucociliary clearance will be impaired, resulting in excessive retention of inhaled particles, microorganisms or endogenous inflammatory debris, and mediating the reactive fibrosis in the bronchoalveolar region, promoting the interstitial lesions (33, 34). Although the mechanisms of MUC5B mediating the interstitial fibrosis are still unknown, it is speculated that it may be related to the injury of ATII cells caused by the excessive MUC5B.

Both the endogenous factors (such as genetics and aging) and environmental factors have been implicated in ATII damages. Infection, drugs, poisons, etc., all cause a certain degree of damages to ATII in ILD with known etiology. In T2DM, the accompanied inflammation is not only closely related to the development of diabetic complications (36), but also could cause the impairment of lung function due to significantly elevated cytokines such as TNF-α, IL-1β and IL-6 (37). However, whether high glucose promotes the development of ILD by causing inflammatory injury of ATII cells needs to be further studied.

In our study, we identified *MUC5B* rs2943512 (A>C) in the pedigree. However, the effects of this SNP on the expression of MUC5B in ATII cells is unclear. The previous study showed that the expression of MUC5B was significantly elevated in pancreatic tissue of patients with T2DM compared to those without T2DM (38), therefore, we speculated that *MUC5B* rs2943512 (A>C) might cause the over-expression of MUC5B in lung tissue of T2DM patients. Even beyond the potential influence of the genetic factor of *MUC5B* SNP rs2943512, whether high glucose itself could cause the over-expression of MUC5B to mediate ATII cells injury and thus trigger pulmonary fibrosis is unclear. Therefore, in order to clarify the relationship between high glucose, MUC5B and ATII injury, this study selected human bronchial epithelial cells BEAS-2B to be stimulated by high glucose in this experiments, to simulate ATII cells (39) in diabetic patients. The results showed that the transcriptional and translational levels of MUC5B in BEAS-2B cells were significantly up-regulated, suggesting that MUC5B existed in the distal airways and ATII cells in the lung tissues of T2DM patients. While this hypothesis needs to be confirmed by *in vivo* experiments in the future.

To explore the potential mechanisms that over-expressed MUC5B promoting fibrosis in alveolar region, we started the experiment from the perspective of inflammatory injury of ATII cells, and detected the cytokines in BEAS-2B cells under high glucose stimulation. The results showed the increased IL-1β and IL-6 was accompanied by the over-expressed MUC5B. Numerous studies have confirmed that IL-1β could cause apoptosis in different types of cell including ATII. Moreover, IL-1β could also play a pro-fibrotic role in certain pathological conditions (40–43). *In vivo* experiments have shown that IL-1β induces progressive pulmonary fibrosis through long-term activation of TGF-β signaling (40). *In vitro*, IL-1β could promote EMT by activating TGF-β in bronchial epithelial cells (44, 45). In addition, IL-1β also stimulates the release of IL-6, which not only play the pro-inflammatory role, but also aggravates the pulmonary fibrosis by activating STAT3 pathway (46).

To clarify the interaction between MUC5B and the cytokines, RNA interference was applied to silence *MUC5B* before BEAS-2B cells were stimulated by high glucose. It was found that IL-1β and IL-6 were significantly decreased in MUC5B knockdown BEAS-2B cells. This finding had rarely been reported in previous studies, and suggested that MUC5B could promote the production of IL-1β and IL-6 in bronchial epithelial cells when stimulated by high glucose, and the over-expressed MUC5B causing inflammatory damage to ATII cells might initiating the pulmonary interstitial fibrosis.

This study also explored the mechanisms of over-expressed MUC5B promoting the synthesis of IL-1β and IL-6 in BEAS-2B cells. It was found that high glucose could cause the activation of ERK1/2 in BEAS-2B cells, while the activation was significantly decreased after *MUC5B* silencing, suggesting that the changes in *MUC5B* transcriptional or translational level might affect ERK1/2 activation, which at least partly explained the changes in IL-1β and IL-6. However, whether MUC5B also affect the synthesis of IL-1β and IL-6 through other pathways remains to be explored. In addition, the molecular mechanisms by which high glucose promotes the over- expression of MUC5B as well as MUC5B promotes the activation of ERK1/2 are both unclear at present, which are needed to be studied in the future.In addition,the lack of further experiments to validate the implications of *MUC5B* site-specific mutation for the functions of BEAS-2B cells and for the aggravation of T2DM and ILD is also one of the limitations of this study. Therefore, we will also focus on the cell and animal experiments related to *MUC5B* site-specific mutation in the next step.

## 5 Conclusion

In this study, a pedigree with T2DM complicated with ILD was selected, and 8 members of the pedigree were included as subjects. Whole-genome resequencing of each subject’s blood was conducted, and the common genetic variants of at least 7 subjects were further screened out, involving numerous SVs, CNVs, SNPs and InDels. The identification of these genetic variants in the pedigree enriches our understanding of the potential genetic contributions to T2DM complicated with ILD. *MUC5B* SNP rs2943512 (A > C) or the up-regulated MUC5B in bronchial epithelial cells may be an important factor in promoting ILD in T2DM patients, making MUC5B a potential biological marker for the development of ILD in diabetic patients, and laying a foundation for future exploration about the pathogenesis of T2DM complicated with ILD.

## Data availability statement

The data presented in the study are deposited in the NCBI repository, accession number PRJNA919498.

## Ethics statement

The studies involving human participants were reviewed and approved by the Ethics Committee of The Second Hospital of Jilin University, China. The patients/participants provided their written informed consent to participate in this study. Written informed consent was obtained from the individual(s) for the publication of any potentially identifiable images or data included in this article.

## Author contributions

QZ and YW collected, analyzed and interpreted the data, drafted and revised the manuscript. CT conducted the cellular experiments, and helped revise the manuscript. JYu and YL helped process and analyze the data. JYa conceived, designed and supervised the study, and guided the revision of the manuscript. All authors read and approved the final manuscript.
